# Hepatitis B virus infection and outcomes of TACE plus lenvatinib and PD-1 inhibitor therapy in unresectable hepatocellular carcinoma: a real-world propensity score-matched study

**DOI:** 10.3389/fimmu.2026.1808956

**Published:** 2026-04-23

**Authors:** Yuhao Su, Yuxin Liang, Deyuan Zhong, Yahui Chen, Shuoshuo Ma, Ming Wang, Qinyan Yang, Hongtao Yan, Xiaolun Huang

**Affiliations:** Liver Transplantation Center and Hepato-Biliary and Pancreatic (HBP) Surgery, Sichuan Cancer Hospital and Institute, Sichuan Cancer Center, School of Medicine, University of Electronic Science and Technology of China, Chengdu, China

**Keywords:** conversion therapy, HBV infection, PD-1 inhibitor, survival outcomes, TACE, tyrosine kinase inhibitor, unresectable HCC

## Abstract

**Background:**

The combination of transarterial chemoembolization (TACE), lenvatinib, and PD-1 inhibitors has shown promising efficacy in treating advanced hepatocellular carcinoma (HCC). However, the impact of hepatitis B virus (HBV) infection on the outcomes of this therapy remains unclear. This study aims to assess the association between HBV infection status and clinical outcomes in patients with initially unresectable HCC undergoing triple therapy.

**Methods:**

This retrospective single-center cohort study included 190 consecutive uHCC patients treated with triple therapy between February 2022 and February 2025. Patients were stratified into HBV-positive (n = 133) and HBV-negative (n = 57) groups based on HBsAg status. Propensity score matching (PSM, 1:1, caliper 0.02) was performed to balance baseline characteristics. The primary endpoint was surgical conversion. Secondary endpoints included tumor response (mRECIST), overall survival (OS), progression-free survival (PFS), and treatment-related adverse events (AEs). Landmark analyses, subgroup analyses and multivariate Cox regression were used to evaluate clinical outcomes and independent prognostic factors.

**Results:**

Compared to HBV-negative patients, HBV-positive patients achieved significantly higher surgical conversion rates both before PSM (25.6% vs. 12.3%, P = 0.041) and after PSM (20% vs. 10%, P = 0.021). After PSM, 30 matched pairs were included. Median OS was not reached in the HBV-positive patients and was 20.0 months in the HBV-negative patients (95% CI, 17.1–22.9; P = 0.014). Median PFS was 28.4 months (95% CI, 25.6–31.2) versus 17.3 months (95% CI, 14.8–19.9; P = 0.030). Tumor response was superior in the HBV-positive group, with higher ORR (36.7% vs. 20%, P = 0.043) and DCR (83.3% vs. 60%, P = 0.045). HBV-positive patients exhibited significantly prolonged OS (P = 0.014) and PFS (P = 0.030). Landmark analysis showed that the PFS advantage in HBV-positive patients no longer statistically significant (P = 0.118). Tumor diameter and HBV infection status were independent predictor of OS and PFS. Grade 3–4 AEs were comparable between groups, and no treatment-related deaths occurred.

**Conclusions:**

HBV-positive status was associated with higher conversion rates and more favorable survival outcomes in unresectable HCC treated with TACE, lenvatinib, and PD-1 inhibitors. Prospective validation is needed to confirm these findings and clarify their biological basis.

## Highlights

- In a real-world uHCC cohort receiving TACE+lenvatinib+PD-1 therapy, HBV-positive status was associated with higher surgical conversion.
- After propensity score matching, conversion remained higher in HBV-positive vs HBV-negative patients (20% vs 10%), with improved ORR and DCR (mRECIST).
- Overall survival favored HBV-positive patients after matching (median not reached vs 20 months); the PFS advantage was attenuated in landmark analysis.
- Baseline maximum tumor diameter and HBV infection status independently predicted both OS and PFS after matching.
- Grade 3–4 adverse events were comparable between groups, with no treatment-related deaths and no HBV reactivation under antiviral management.

## 1 Introduction

Hepatocellular carcinoma (HCC) is the predominant form of primary liver cancer and a major global health challenge (1). It accounts for roughly 90% of liver cancer cases and is one of the leading causes of cancer-related mortality worldwide, responsible for over 800,000 deaths annually (2–4). The geographic distribution of HCC is highly uneven: incidence is particularly high in East Asia, Southeast Asia, and sub-Saharan Africa, often exceeding 40 cases per 100,000 population in the most affected countries (2–4). Overall, global incidence trends have been rising due to population aging and growth, despite some declines in settings with effective HBV vaccination and antiviral treatment programs (3–5). Five-year survival remains poor (often below 20%), reflecting that most patients are diagnosed at advanced stages with limited curative options (1, 6).

Chronic hepatitis B virus (HBV) infection is a well-established etiological factor in HCC (1, 3). In regions where HBV is endemic, such as much of Asia and Africa, the majority of HCC cases are attributable to long-standing HBV infection (3). Persistent HBV promotes liver carcinogenesis through multiple mechanisms, including ongoing inflammation and cirrhosis, integration of viral DNA into the host genome, and expression of viral oncoproteins that drive oncogenic signaling (7, 8). Globally, it is estimated that over 250 million people are chronically infected with HBV, the largest reservoir of HBV-related liver cancer risk (9). Vaccination of newborns against HBV and effective antiviral therapy have begun to reduce HBV prevalence in younger cohorts, but HBV remains a dominant risk factor for HCC in many countries (5, 9).

For patients with intermediate to advanced HCC that is initially unresectable, a triple-combination conversion approach incorporating transarterial chemoembolization (TACE), molecular targeted tyrosine kinase inhibition (TKI), and programmed cell death protein-1 (PD-1) immune checkpoint blockade has emerged as a promising strategy (10, 11). Early clinical reports suggest that this multimodal approach can achieve high objective response rates and convert a significant proportion of unresectable tumors to resectable, indicating its potential as an effective downstaging strategy (11). Despite the promise of triple conversion therapy, a critical uncertainty remains: it is not known whether underlying HBV infection status influences treatment efficacy. Most clinical studies of this combination involve patient populations with a high prevalence of chronic HBV, but few have stratified outcomes by viral status. Chronic HBV infection can alter the immune microenvironment in the liver, which could affect response to targeted and immune therapies (12). Conversely, immune checkpoint inhibition in HBV-positive patients may carry risks of viral reactivation or immune-mediated hepatic toxicity (13, 14). Together, these considerations underscore that the net impact of HBV on triple therapy outcomes is unclear.

This study used a propensity score-matched design to investigate whether HBV serostatus was associated with tumor response, survival outcomes, and conversion rate in patients receiving triple conversion therapy. By treating HBV status as a clinical covariate rather than implying causation, the analysis will identify whether HBV status is associated with therapeutic outcomes. Identifying such associations may inform patient stratification and antiviral management, with the ultimate goal of optimizing therapeutic strategies for virus-related HCC. This case-control study has been reported in line with the STROCSS guidelines (15).

## 2 Research objects and methods

### 2.1 Study design and methods

This retrospective cohort study included consecutive patients diagnosed with unresectable HCC (uHCC) between February 2022 and February 2025 at Sichuan Cancer Hospital and Sichuan Provincial People’s Hospital. All patients received triple-combination therapy consisting of TACE, lenvatinib, and PD-1 inhibitors, administered by either the hepatobiliary surgery or interventional radiology departments.

Inclusion criteria were as follows: (1) age 18–75 years; (2) HCC confirmed pathologically or radiologically according to the Chinese Guidelines for the Diagnosis and Treatment of Primary Liver Cancer; (3) initial assessment as unresectable by a multidisciplinary team based on tumor location, multiplicity, size, vascular invasion, or insufficient future liver remnant volume; (4) receipt of at least one cycle of triple therapy (TACE + lenvatinib + a PD-1 inhibitor including sintilimab, atezolizumab, camrelizumab, or pembrolizumab); (5) availability of complete baseline and follow-up data. Exclusion criteria included: (1) concurrent other malignancies; (2) severe liver dysfunction (Child–Pugh class C) or extrahepatic organ failure; (3) severe autoimmune diseases or long-term immunosuppressive therapy; (4) coinfection with HIV or hepatitis C virus; (5) incomplete clinical records or loss to follow-up.

A total of 190 patients met the eligibility criteria, comprising 133 HBV-positive and 57 HBV-negative patients based on hepatitis B surface antigen (HBsAg) status. Written informed consent forms for the biological sample library have been obtained from all participants prior to enrollment.

### 2.2 Treatment protocol

All patients received standard triple therapy. All patients received a standardized transarterial chemoembolization protocol. Following vascular catheterization, a microcatheter was superselectively advanced into the tumor-feeding arteries. Chemotherapeutic agents, most commonly lobaplatin at a dose of 50 to 100 mg, were administered via intra-arterial infusion, followed by delivery of an iodized oil emulsion in a volume ranging from 5 to 20 mL. Subsequent embolization was performed using gelatin sponge particles or polyvinyl alcohol microspheres until complete disappearance of tumor staining was achieved on angiography. Lenvatinib was initiated within one week following TACE. PD-1 inhibitors, including sintilimab (200 mg), camrelizumab (200 mg), atezolizumab (1200 mg), or pembrolizumab (200 mg) were administered intravenously every three weeks, beginning concurrently with targeted therapy after the initial TACE session. Treatment cycles were determined by tumor response and patient tolerance. Radiological assessment was performed every 6–8 weeks to guide subsequent TACE sessions. Patients who achieved downstaging and satisfied criteria for resection were evaluated for radical hepatectomy.

Tumor resectability was evaluated by a multidisciplinary team based on radiologic response and clinical status. Surgical eligibility was defined by the following criteria: (1) preserved liver function and general condition, indicated by a Child–Pugh score ≤ 7, indocyanine green retention rate at 15 minutes (ICG R15) ≤ 10%, and ECOG performance status of 0–1; (2) complete response (CR) and partial response (PR), or stable disease (SD) maintained for at least 2 months, provided all other resectability criteria are met according to modified RECIST (mRECIST); (3) sufficient future liver remnant volume, defined as ≥ 35% of standard liver volume in patients without cirrhosis and ≥ 45% in those with cirrhosis; (4) regression or inactivation of vascular tumor thrombi with technical feasibility for curative resection and an intent to achieve R0 margins; and (5) absence of other contraindications to surgery. The detailed MDT decision template is provided in **Supplementary Table 1**.

All patients who tested positive for hepatitis B surface antigen underwent assessment of HBV DNA levels at initial hospital admission. Antiviral therapy was initiated and adjusted in accordance with the virological results. Prior to initiation of triple-combination therapy, viral replication was adequately controlled in all affected patients, with HBV DNA levels below 100 IU/mL. During conversion therapy, HBV DNA levels were monitored longitudinally.

### 2.3 Data collection and outcome measures

Baseline demographic and clinical variables were collected, including age, sex, Child–Pugh classification, Barcelona Clinic Liver Cancer (BCLC) stage, maximum tumor diameter, tumor number, total bilirubin, alpha-fetoprotein (AFP) level, albumin, C-reactive protein (CRP), cirrhosis status, portal vein tumor thrombus (PVTT), and antiviral therapy status in HBV-positive patients.

The primary endpoint was the conversion rate to curative surgery, defined as the proportion of patients who achieved sufficient tumor downstaging to enable R0 resection following conversion therapy. Secondary endpoints included tumor response, progression-free survival (PFS), overall survival (OS), safety. Tumor response, evaluated according to modified Response Evaluation Criteria in Solid Tumors (mRECIST) criteria (16) including complete response (CR), partial response (PR), stable disease (SD), progressive disease (PD), objective response rate (ORR = CR + PR), and disease control rate (DCR = CR + PR + SD). PFS was defined as the time from treatment initiation to radiologic progression, postoperative recurrence, or death from any cause, whichever occurred first. OS was defined as the time from treatment initiation to death from any cause. Safety was evaluated by monitoring treatment-related adverse events (TRAEs), graded according to the Common Terminology Criteria for Adverse Events version 5.0 (17).

### 2.4 Statistical analysis

Continuous variables were expressed as mean ± standard deviation or median (interquartile range) and compared using independent-sample t-tests or Mann–Whitney U tests, as appropriate. Categorical variables were summarized as frequencies (%) and compared using chi-square or Fisher’s exact tests.

To minimize confounding from baseline imbalances, 1:1 propensity score matching (PSM) was applied using the nearest-neighbor method with a caliper of 0.02. The propensity score model included covariates such as sex, age, BCLC stage, ECOG performance status, maximum tumor diameter, and AFP level, and the propensity scores were calculated using logistic regression (18, 19). After matching, the Kaplan-Meier method was used to evaluate survival, whereas the reverse Kaplan-Meier method was used to estimate median follow-up time. The differences were evaluated with the log-rank test. The Cox proportional hazards model was used to identify prognostic factors for survival. It was used to estimate hazard ratios (HRs) and 95% confidence intervals (CIs).

To evaluate the association between clinical complete response (cCR) and survival in the entire cohort, landmark analysis was employed to address immortal time bias. For the OS analysis, patients who died or were lost to follow-up before the landmark time were excluded. Similarly, for the PFS analysis, patients who had experienced disease progression or were lost to follow-up before the landmark time were excluded. Only patients achieving cCR by the landmark time were classified as cCR. Furthermore, the landmark times were set at 5 and 6 months, corresponding to the points at which 75% and 90% of patients had achieved cCR, respectively. In the analysis of patients who underwent conversion surgery, cCR status had been determined prior to surgery, thereby eliminating the concern for immortal time bias. A two-sided P value < 0.05 was considered statistically significant. All statistical analyses were conducted using SPSS software (version 17.0; SPSS Inc., Chicago, IL, USA) and R (version 4.3.3).

## 3 Research results

### 3.1 Patient baseline characteristics

**Figure 1:**
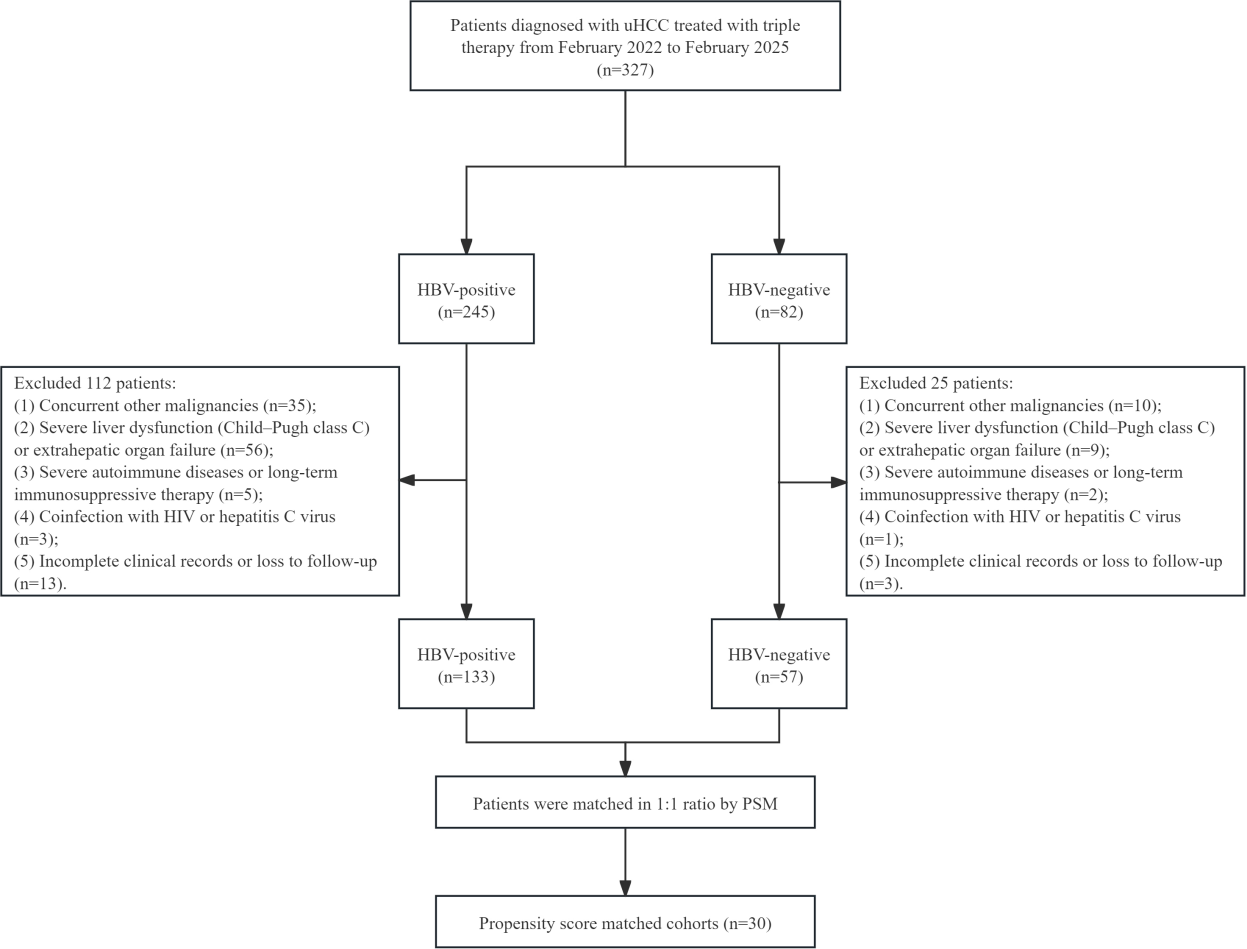
Patient enrollment flowchart. uHCC, unresectable hepatocellular carcinoma; HBV, Hepatitis B Virus; HIV, Human Immunodeficiency Virus; PSM, propensity score matching.

A total of 190 patients with unresectable HCC who received triple-combination therapy were included in the analysis, comprising 133 HBV-positive group and 57 HBV-negative group (**Figure 1**). Before PSM, HBV-positive patients had significantly higher baseline AFP levels compared with HBV-negative patients (P < 0.05). After 1:1 PSM, 30 matched pairs were successfully created. Post-matching, there were no statistically significant differences between the two groups in all key baseline characteristics, including age, gender, Child-Pugh grade, BCLC stage, tumor burden, AFP level, and portal vein tumor thrombus status (P > 0.05), indicating good balance. We analyzed the baseline antiviral treatment status and baseline HBV-DNA levels in the HBV-positive group, as well as the baseline etiologic distribution in the HBV-negative group, to provide a more intuitive presentation of the underlying etiology distribution (**Table 1**).

**Table 1:** Patient characteristics before and after propensity score matching.

Variables	Before PSM			After PSM		
	HBV-positive	HBV-negative	P value	HBV-positive	HBV-negative	P value
	N=133	N=57		N=30	N=30	
age (years), n (%)			0.306			0.573
≥65	40 (30.1%)	13 (22.8%)		10 (33.3%)	8 (26.7%)	
<65	93 (69.9%)	44 (77.2%)		20 (66.7%)	22 (73.3%)	
gender, n (%)			0.445			0.781
Male	96 (72.2%)	38 (66.7%)		20 (66.7%)	21 (70.0%)	
Female	37 (27.8%)	19 (33.3%)		10 (33.3%)	9 (30.0%)	
ECOG score, n (%)			0.680			0.436
0	68 (51.1%)	31 (54.4%)		12 (40.0%)	15 (50.0%)	
1	65 (48.9%)	26 (45.6%)		18 (60.0%)	15 (50.0%)	
BCLC stage, n (%)			0.174			0.542
C	51 (38.3%)	16 (28.1%)		24 (80.0%)	22 (73.3%)	
B	82 (61.7%)	41 (71.9%)		6 (20.0%)	8 (26.7%)	
Child–Pugh score, n (%)			0.173			0.390
B	46 (34.6%)	14 (24.6%)		20 (66.7%)	23 (76.7%)	
A	87 (65.4%)	43 (75.4%)		10 (33.3%)	7 (23.3%)	
AFP (ng/ml), n (%)			**0.048**			0.222
<400	92 (69.2%)	47 (82.5%)		25 (83.3%)	21 (70.0%)	
≥400	41 (30.8%)	10 (17.5%)		5 (16.7%)	9 (30.0%)	
tumor diameter (cm), n (%)			0.849			0.071
≥7	68 (51.1%)	30 (52.6%)		11 (36.6%)	18 (60.0%)	
<7	65 (48.9%)	27 (47.4%)		19 (63.4%)	12 (40.0%)	
tumor number, n (%)			0.460			0.432
<3	53 (39.8%)	26 (45.6%)		11 (36.6%)	14 (46.6%)	
≥3	80 (60.2%)	31 (54.4%)		19 (63.4%)	16 (53.4%)	
TBIL, median (IQR)	18.6 (12.8, 23.1)	15.5 (9.8, 22.9)	0.058	18.2 (11.975, 22.925)	14.15 (9.825, 19.625)	0.152
CRP, median (IQR)	4.57 (1.92, 10.59)	4.59 (1.63, 15.36)	0.975	4.19 (1.3425, 16.252)	3.17 (0.9775, 14.603)	0.773
ALB, median (IQR)	37.9 (33.7, 40.4)	38.6 (35.2, 41)	0.473	37.75 (35.325, 39.55)	38.65 (34.225, 41.075)	0.589
cirrhosis, n (%)			0.356			0.371
Absence	44 (33.1%)	15 (26.3%)		6 (20.0%)	9 (30.0%)	
Presence	89 (66.9%)	42 (73.7%)		24 (80.0%)	21 (70.0%)	
PVTT, n (%)			0.174			0.542
Presence	51 (38.3%)	16 (28.1%)		24 (80.0%)	22 (73.3%)	
Absence	82 (61.7%)	41 (71.9%)		6 (20.0%)	8 (26.7%)	
Conversion, n (%)			0.041			0.021
Presence	34 (25.6%)	7 (12.3%)		6 (20.0%)	3 (10.0%)	
Absence	99 (74.4%)	50 (87.7%)		24 (80.0%)	27 (90.0%)	
Progression, n (%)			< 0.001			0.055
Presence	26 (19.5%)	26 (45.6%)		5 (16.7%)	12 (40.0%)	
Absence	108 (80.5%)	31 (54.4%)		25 (83.3%)	18 (60.0%)	
Death, n (%)			0.011			0.118
Presence	44 (33.1%)	30 (52.6%)		10 (33.3%)	16 (53.3%)	
Absence	89 (66.9%)	27 (47.4%)		20 (66.7%)	14 (46.7%)	
Etiology, n (%)			NA			NA
HBV	133 (100.0%)	NA		30 (100.0%)	NA	
MASLD	NA	12 (21.1%)		NA	6 (20.0%)	
HCV	NA	9 (15.8%)		NA	5 (16.7%)	
Others	NA	36 (63.1%)		NA	19 (63.3%)	
Antiviral therapy, n (%)			NA			NA
ETV	28 (21.1%)	NA		6 (20.0%)	NA	
TDF	105 (78.9%)	NA		24 (80.0%)	NA	
HBV-DNA (IU/mL), n (%)			NA			NA
≥100	0	NA		0	NA	
<100	133 (100.0%)	NA		30 (100.0%)	NA	

PSM, propensity score matching; HBV, hepatitis B virus; AFP, alpha-fetoprotein; BCLC, Barcelona Clinic Liver Cancer; PVTT, portal vein tumor thrombus; ECOG, Eastern Cooperative Oncology Group; OS, overall survival; PFS, Progression-free survival; ALB, albumin; TBIL, total bilirubin; CRP, C-reactive protein; HCV, hepatitis C virus; MASLD, Metabolic Dysfunction-Associated Steatotic Liver Disease; ETV, Entecavir; TDF, Tenofovir Disoproxil Fumarate; NA, not applicable. Bold values indicate statistical significance at P < 0.05.

### 3.2 Surgical conversion

Regarding the primary outcome, before PSM, 34 patients in HBV-positive group successfully achieved surgical conversion and underwent radical resection, with a conversion rate of 25.6% (34/133). In contrast, only 7 patients in HBV-negative group achieved conversion, with a rate of 12.3% (7/57). This difference was statistically significant (P = 0.041). After PSM, 6 patients in HBV-positive group achieved surgical conversion, with a rate of 20% (6/30), compared to 3 patients in HBV-negative group, with a rate of 10% (3/30). The difference remained statistically significant (P = 0.021).

### 3.3 Tumor response

Regarding tumor response, assessed by mRECIST criteria post-PSM, HBV-positive group also showed significantly better efficacy than HBV-negative group. The ORR of HBV-positive group reached 36.7% (11/30, including 6 CR and 5 PR), significantly higher than 20% in HBV-negative group (6/30, including 2 CR and 4 PR) (P = 0.043). The DCR of HBV-positive group was 83.3% (25/30), also significantly higher than 60% in HBV-negative group (18/30) (P = 0.045). Detailed tumor response data for both groups are shown in **Table 2**.

**Table 2:** Tumor response based on the modified response evaluation criteria in solid tumors.

Variables	Before PSM			After PSM		
	HBV-positive	HBV-negative	P value	HBV-positive	HBV-negative	P value
CR	12 (9.0%)	2 (3.5%)	0.003	6 (20%)	2 (6.7%)	0.035
PR	78 (58.6%)	23 (40.4%)		5 (16.7%)	4 (13.3%)	
SD	17 (12.8%)	6 (10.5%)		14 (46.6%)	12 (40%)	
PD	26 (19.6%)	26 (45.6%)		5 (16.7%)	12 (40%)	
ORR	90 (67.7%)	25 (43.9%)	0.002	11 (36.7%)	6 (20%)	0.043
DCR	107 (80.5%)	31 (54.4%)	<0.001	25 (83.3%)	18 (60%)	0.045

PSM, propensity score matching; HBV, hepatitis B virus; CR, complete response; PR, partial response; SD, stable disease; PD, progressive disease; ORR, objective response rate; DCR, disease control rate.

### 3.4 Survival analysis

**Figure 2:**
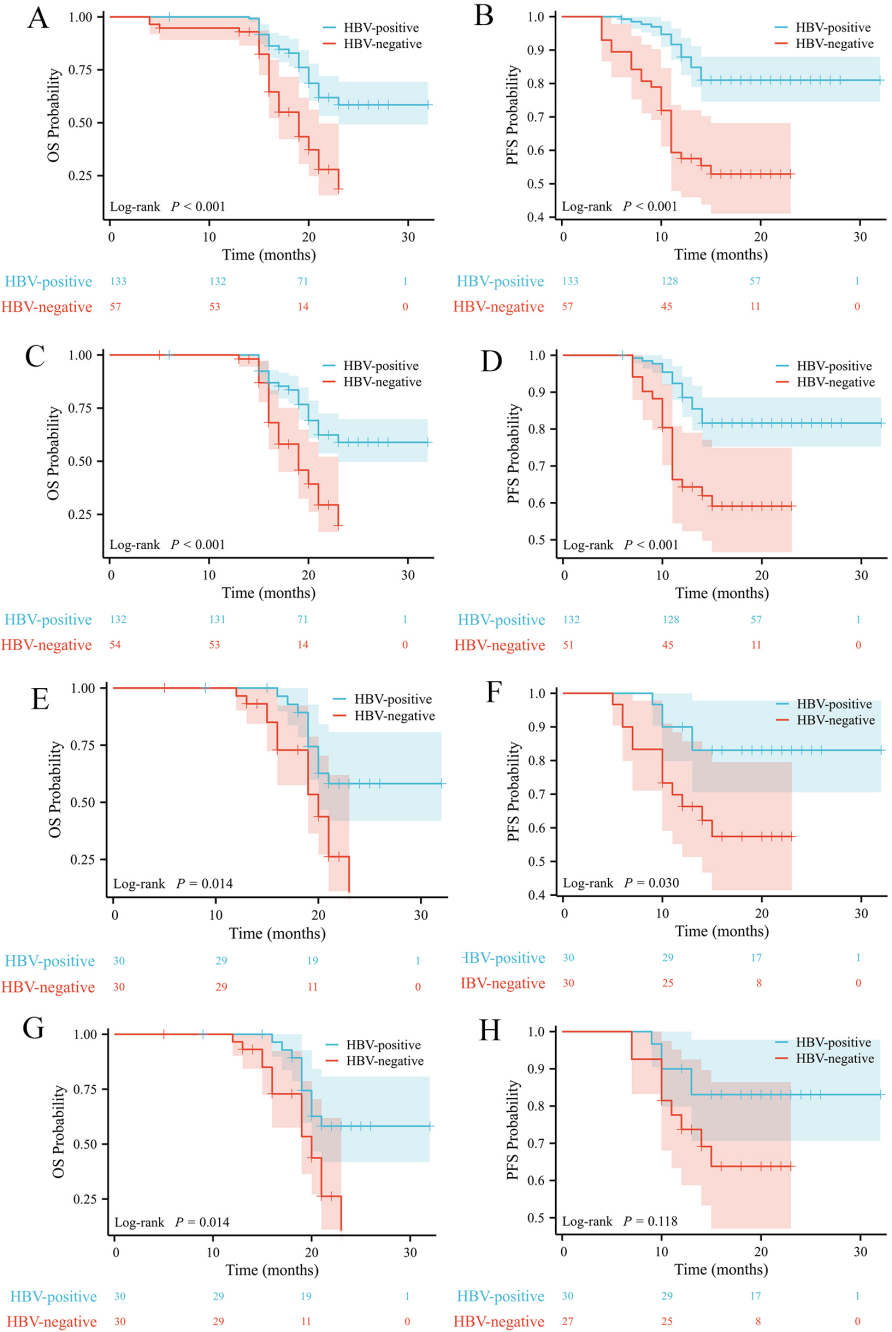
Kaplan–Meier survival analysis and landmark analysis for OS and PFS before PSM and after PSM. Kaplan–Meier survival analysis for OS **(A)** and PFS **(B)** before PSM; OS **(E)** and PFS **(F)** after PSM. Landmark analysis for OS **(C)** and PFS **(D)** before PSM; OS **(G)** and PFS **(H)** after PSM. OS, overall survival; PFS, Progression-free survival; HBV, Hepatitis B Virus; PSM, propensity score matching.

The median follow-up time was 19.0 months. Before PSM, 44 deaths occurred in HBV-positive group (33.1%) and 30 in HBV-negative group (52.6%). The median OS for HBV-positive group was not reached, while it was 19 months for HBV-negative group (95% CI: 16.8-21.2) (P < 0.001) (**Figure 2A**). After PSM, 10 deaths occurred in HBV-positive group (33.3%) and 16 in HBV-negative group (53.3%). The median OS for HBV-positive group was not reached, while it was 20 months for HBV-negative group (95% CI: 17.1-22.9) (P = 0.014) (**Figure 2E**). Before PSM, 26 disease progressions occurred in HBV-positive group (19.5%) and 26 in HBV-negative group (45.6%). The median PFS was 28.1 months for HBV-positive group (95% CI: 26.7-29.5) and 16.6 months for HBV-negative group (95% CI: 14.7-18.4) (P < 0.001)(**Figure 2B**). After PSM, 5 progressions occurred in HBV-positive group (16.7%) and 12 in HBV-negative group (40%). The median PFS was 28.4 months for HBV-positive group (95% CI: 25.6-31.2) and 17.3 months for HBV-negative group (95% CI: 14.8-19.9) (P = 0.030) (**Figure 2F**). In landmark analyses conducted before and after PSM, significant differences in both OS and PFS were observed between HBV-positive and HBV-negative patients in the unmatched cohort (both P < 0.001)(**Figures 2C, D**). Following PSM, the survival advantage associated with HBV positivity persisted for OS but was attenuated for PFS, which did not reach statistical significance (P = 0.118)(**Figures 2G, H**). After PSM, multivariate analysis identified maximum tumor diameter (OS: HR = 0.536; 95% CI, 0.294-1.237; P = 0.019; PFS: HR = 0.145; 95% CI, 0.032-0.650; P = 0.012) and HBV infection status (OS: HR = 3.511; 95% CI, 1.557-7.919; P = 0.002; PFS: HR = 6.355; 95% CI, 2.174-18.575; P<0.001) as an independent predictive factor for both OS and PFS (**Table 3**).

**Table 3:** Predictive factor analysis for progression-free survival and overall survival (after matching).

Characteristics	PFS				OS			
	Univariate analysis		Multivariate analysis		Univariate analysis		Multivariate analysis	
	HR (95% CI)	P value	HR (95% CI)	P value	HR (95% CI)	P value	HR (95% CI)	P value
Age, years (<65 vs. ≥65)	0.538 (0.204 - 1.415)	0.209			0.375 (0.167 - 0.839)	**0.017**	0.358 (0.157 - 0.820)	**0.015**
Gender (Male vs. Female0	0.670 (0.218 - 2.055)	0.484			0.701 (0.281 - 1.745))	0.445		
ECOG score (1 vs. 0)	1.208 (0.460 - 3.175)	0.701			0.704 (0.326 - 1.520)	0.371		
BCLC stage (B vs. C)	0.377 (0.086 - 1.648)	0.195			0.691 (0.237 - 2.012)	0.498		
Child-Pugh score (A vs. B)	0.758 (0.247 - 2.327)	0.628			0.520 (0.202 - 1.339)	0.176		
AFP, ng/mL (<400 vs. ≥400)	1.410 (0.497 - 4.005)	0.518			1.598 (0.710 - 3.596)	0.258		
TBIL, μmol/L (< 17.1 vs. ≥17.1)	0.877 (0.338 - 2.273)	0.787			0.904 (0.417 - 1.957)	0.797		
CRP, mg/L (<10 vs. ≥10)	0.838 (0.295 - 2.381)	0.741			0.762 (0.317 - 1.830)	0.543		
ALB, g/L (<35 vs. ≥35)	0.863 (0.281 - 2.647)	0.796			0.916 (0.366 - 2.292)	0.850		
Tumor diameter, cm (<7 vs. ≥7)	0.114 (0.026 - 0.500)	**0.004**	0.145 (0.032 - 0.650)	**0.012**	0.479 (0.207 - 1.107)	**0.045**	0.536 (0.294– 1.237)	**0.019**
Tumor number, (<3 vs. ≥3)	0.000 (0.000 - Inf)	0.997			0.000 (0.000 - Inf)	0.997		
Cirrhosis (No vs. Yes)	0.590 (0.169 - 2.052)	0.406			0.593 (0.204 - 1.723)	0.337		
HBV status (No vs. Yes)	2.988 (1.050 - 8.502)	**0.04**	6.355 (2.174 - 18.575)	**<0.001**	2.824 (1.264 - 6.306)	**0.011**	3.511 (1.557 - 7.919)	**0.002**

PFS, progression-free survival; OS, overall survival; HR, hazard ratio; CI, confidence interval; AFP, alpha-fetoprotein; BCLC, Barcelona Clinic Liver Cancer; ECOG, Eastern Cooperative Oncology Group; TBIL, total bilirubin; CRP, C-reactive protein; ALB, albumin; HBV, Hepatitis B Virus. Bold values indicate statistical signific ance at P < 0.05.

### 3.5 Subgroup analysis

**Figure 3:**
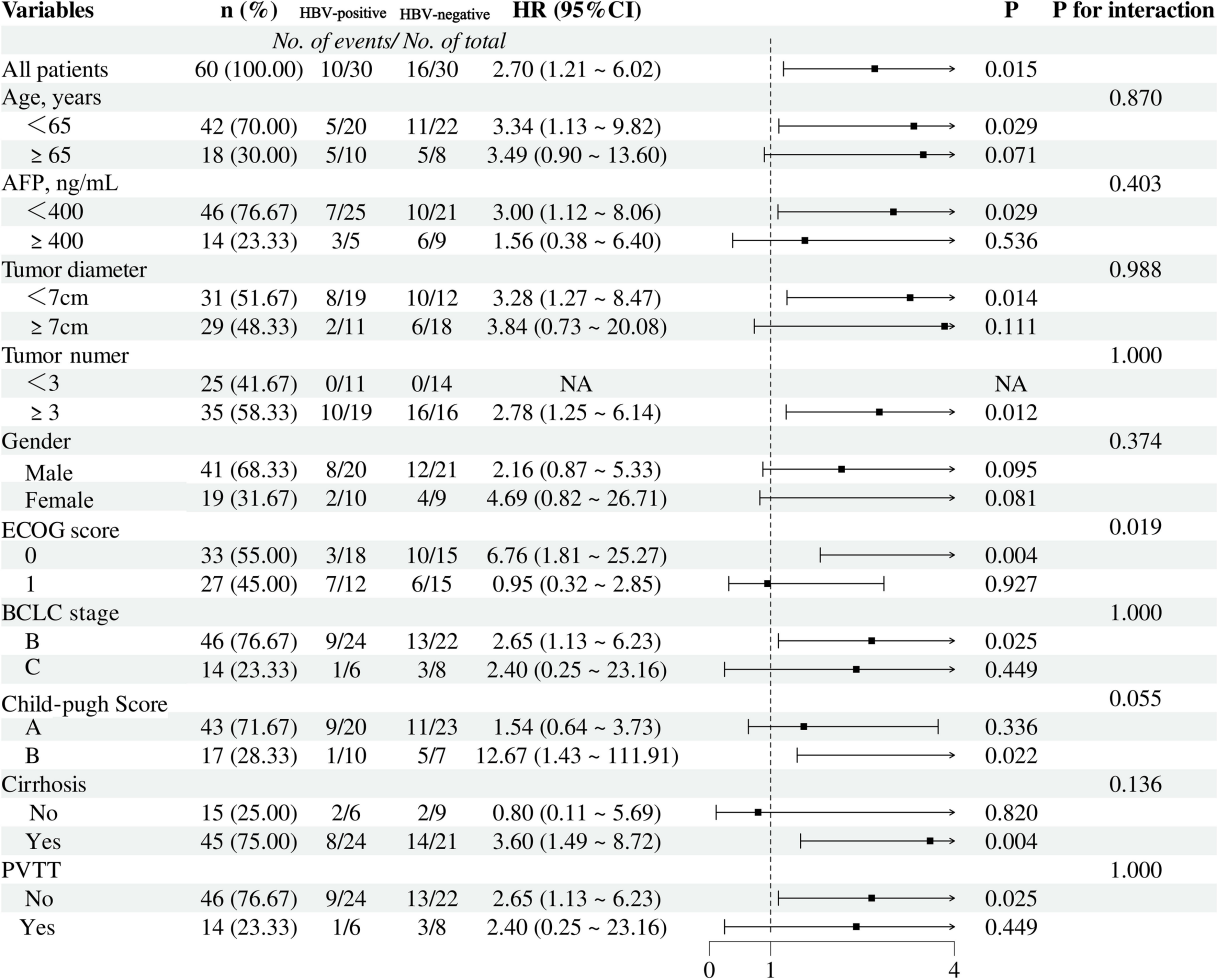
Forest plot for overall survival of the two groups of patients after PSM. HR, hazard ratio; AFP, alpha-fetoprotein; ECOG, Eastern Cooperative Oncology Group; BCLC stage, Barcelona Clinic liver cancer stage; PVTT, portal vein tumor thrombus.

**Figure 4:**
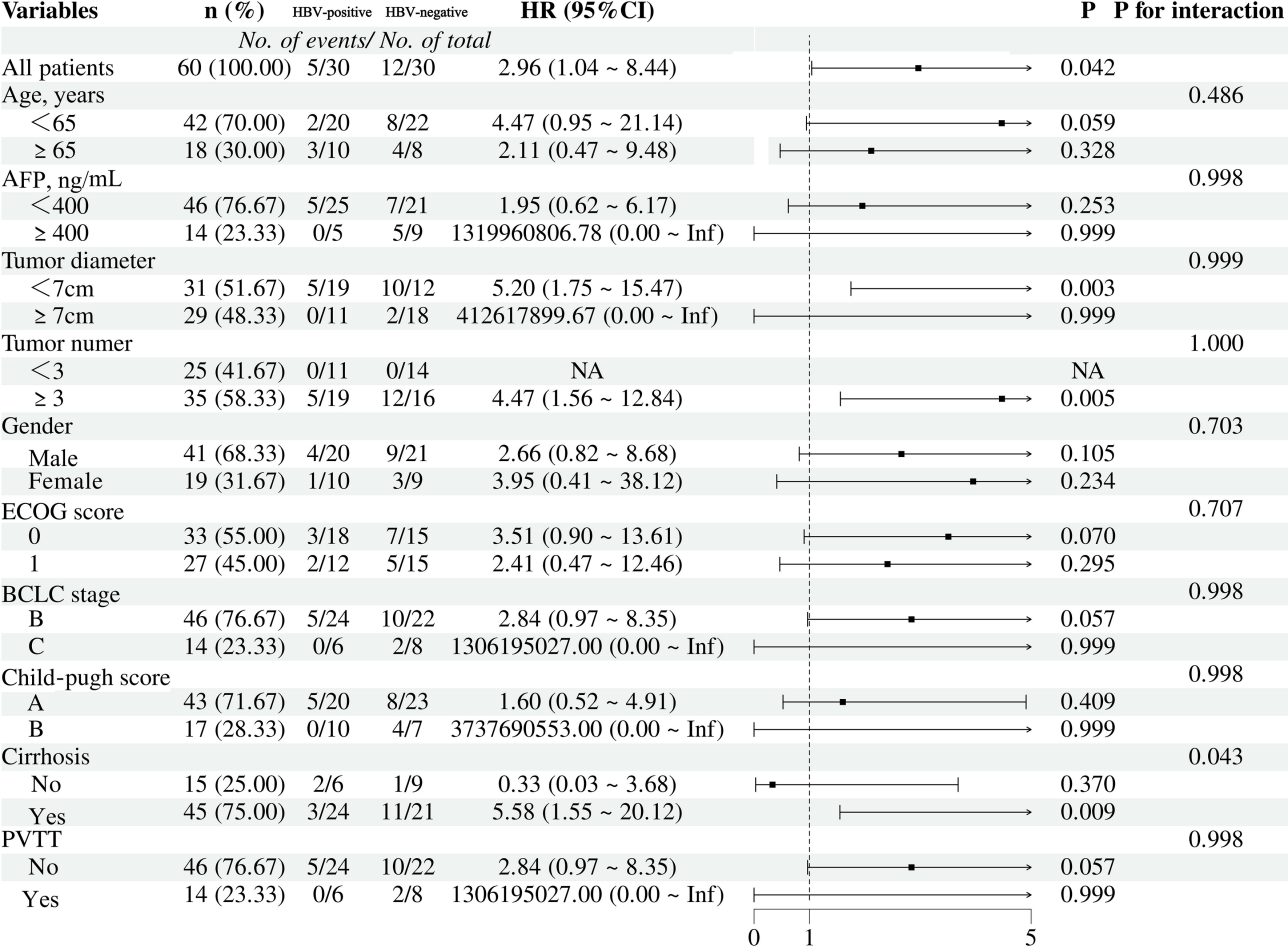
Forest plot for progression-free survival of the two groups of patients after PSM. HR, hazard ratio; AFP, alpha-fetoprotein; ECOG, Eastern Cooperative Oncology Group; BCLC stage, Barcelona Clinic liver cancer stage; PVTT, portal vein tumor thrombus.

Subgroup analyses of factors associated with OS and PFS after PSM are shown as forest plots in **Figures 3**, **4**. In the overall cohort receiving triple-combination conversion therapy, HBV-negative patients had significantly worse OS (HR = 2.70, 95% CI 1.21–6.02; P = 0.015) and PFS (HR = 2.96, 95% CI 1.04–8.44; P = 0.042) than HBV-positive patients. Subgroup analyses for OS and PFS indicated that the association between HBV status and survival outcomes was generally consistent across most predefined clinical and tumor-related strata, with no evidence of effect heterogeneity (P for interaction > 0.05). Notably, a significant interaction was observed with Eastern Cooperative Oncology Group (ECOG) performance status (P for interaction = 0.019): among patients with ECOG 0, HBV negativity was associated with a markedly increased risk of death (HR = 6.76, 95% CI 1.81–25.27; P = 0.004), whereas no significant difference was detected in those with ECOG 1 (HR = 0.95, 95% CI 0.32–2.85; P = 0.927). In addition, in the PFS subgroup analysis, cirrhosis showed a significant interaction with HBV status (P for interaction = 0.043): HBV-negative patients with cirrhosis were more likely to experience disease progression (HR = 5.58, 95% CI 1.55–20.12; P = 0.009), while no significant difference was observed among patients without cirrhosis (HR = 0.33, 95% CI 0.03–3.68; P = 0.370).

### 3.6 Adverse events

Treatment-related adverse events are listed in **Table 4**. There was no statistically significant difference in the incidence of adverse events between the HBV-positive group and HBV-negative group. These adverse events resolved or were eliminated after conservative treatment, and no treatment-related deaths occurred. Additionally, in the HBV-positive group, 33.3% (10/30) of patients required dose reduction or temporary interruption of targeted drugs. One patient in the HBV-positive group interrupted treatment due to hypothyroidism. After receiving symptomatic treatment, the patient resumed the previous treatment regimen. Throughout the conversion therapy period, longitudinal monitoring of HBV DNA levels was performed in hepatitis B virus positive patients, and no cases of hepatitis B virus reactivation were observed.

**Table 4:** Treatment-related adverse events (after matching).

Events, n (%)	HBV-positive (n=30)			HBV-negative (n=30)			P value		
	Any grade	Grade 1/2	Grade 3/4	Any grade	Grade 1/2	Grade 3/4	Any grade	Grade 1/2	Grade 3/4
Any TRAE	22	22	7	23	23	5	0.766	0.766	0.748
Hematologic toxic effects									
Thrombocytopenia	3	2	1	1	1	0	0.605	>0.999	>0.999
Leukopenia	2	2	0	1	1	0	>0.999	>0.999	>0.999
Hepatic function									
Elevated AST	5	4	1	4	3	1	>0.999	>0.999	>0.999
Elevated ALT	5	4	1	4	3	1	>0.999	>0.999	>0.999
Hyperbilirubinemia	6	5	1	5	4	1	>0.999	>0.999	>0.999
Hypertension	2	2	0	4	4	0	0.667	0.667	>0.999
Non-hematologic toxic effects									
Nausea	7	7	0	6	6	0	>0.999	>0.999	>0.999
Fatigue	5	4	1	6	4	2	>0.999	>0.999	>0.999
Fever	3	2	1	4	3	1	>0.999	>0.999	>0.999
Pain	1	1	0	2	1	1	>0.999	>0.999	>0.999
Diarrhea	2	1	1	1	1	0	>0.999	>0.999	>0.999
Hand-foot-skin reactions	2	2	0	1	1	0	>0.999	>0.999	>0.999
Hypertension	3	2	1	2	2	0	>0.999	>0.999	>0.999
Hypothyroidism	1	0	1	0	0	0	>0.999	>0.999	>0.999

HBV, hepatitis B virus; TRAE, Treatment-related adverse event; AST, aspartate transaminase; ALT, alanine aminotransferase.

## 4 Discussion

In this cohort of patients with initially uHCC treated with a triple-combination conversion strategy, we explored whether HBV status is associated with differential treatment performance in a real-world setting. After PSM, HBV-positive patients showed a higher probability of reaching surgical conversion (approximately 20% vs 10%) and achieved numerically better radiologic tumor control, with higher ORR (36.7% vs 20%) and DCR (83.3% vs 60%). Survival curves likewise separated in favor of the HBV-positive group, with median OS not reached and a longer median PFS (about 28.4 vs 17.3 months). In the landmark analysis performed in the PSM cohort, the PFS advantage observed in HBV-positive patients was no longer statistically significant when compared with HBV-negative patients. This attenuation may be partly attributable to the reduced analyzable sample size after matching, which inevitably limited statistical power and may have precluded the detection of modest between-group differences. Importantly, the frequency of grade 3 to 4 adverse events was comparable between groups and no treatment-related deaths occurred, suggesting that the observed efficacy differences were not accompanied by an obvious safety trade-off. Collectively, these findings suggest that HBV status may be associated with differences in treatment outcomes in the setting of TACE-based triple therapy. Although HBV status may have potential value as a stratification factor when evaluating candidates for conversion treatment, this possibility remains hypothesis-generating and should be validated in prospective studies before clinical application.

The biological basis of HBV-associated heterogeneity remains incompletely understood. Chronic HBV infection is associated with persistent antigen exposure, ongoing inflammation, and exhausted CD8^+^ T cells with upregulated inhibitory checkpoints, including PD-1, TIM-3, and LAG-3 (12, 20). However, some of these exhausted T cells may still be reinvigorated by PD-1 blockade (21). At the same time, HBV-related tumors may also retain immunosuppressive features that limit durable tumor control (12, 22). Therefore, HBV status may be better interpreted as a surrogate of a broader immune context rather than a direct determinant of treatment benefit (22, 23). In our study, the higher DCR observed in HBV-positive patients differed from reports showing broadly similar responses to ICI monotherapy across viral and non-viral HCC etiologies (23–26). One possible explanation is that the addition of locoregional and antiangiogenic therapy may enhance antigen priming and partially overcome immune suppression, thereby making etiology-related differences more clinically apparent (22, 24, 27). HBV infection may also contribute to enhanced angiogenic signaling in HCC (8, 28). HBV-related proteins can upregulate VEGF and related pathways, while lenvatinib may counteract these effects by inhibiting VEGFR/FGFR signaling, improving vascular normalization, and enhancing immune infiltration (8, 28–32). Experimental studies further suggest that lenvatinib may modulate the immune microenvironment by increasing CD8+ T-cell infiltration and reducing immunosuppressive features (33–36). In this context, HBV status may reflect differences in angiogenic-immune interactions relevant to combination therapy response, although a causal relationship cannot be inferred. TACE may further strengthen antitumor immunity by inducing immunogenic cell death and promoting the release of tumor antigens and danger signals (27, 37, 38). These changes may enhance antigen presentation and immune cell recruitment, and such effects could be more relevant in HBV-positive patients with pre-existing virus-related antigen exposure (22, 27, 37, 39, 40). Collectively, our findings suggest that the efficacy of triple therapy may vary by disease etiology, and that HBV status may serve as a population-level marker of this variability rather than indicating uniform benefit in all HBV-positive patients (22, 23).

Published prospective studies have established TACE-based triple therapy as an active conversion strategy for unresectable HCC (41, 42). Against this background, our findings are directionally consistent with the reported antitumor activity of this regimen, but address a different question: whether HBV status is associated with heterogeneity in conversion and survival outcomes within an already active treatment platform. Clinically, HBV status may help refine baseline stratification when considering the likelihood of conversion and disease control, although this should be interpreted cautiously. Importantly, safety was comparable between HBV-positive and HBV-negative patients, consistent with the manageable toxicity reported for lenvatinib- and PD-1-based combination regimens in real-world practice (10, 43). Thus, the main contribution of this study is not to reconfirm the efficacy of triple therapy itself, but to suggest that HBV status may capture clinically relevant heterogeneity in treatment benefit, a possibility that remains hypothesis-generating until prospectively validated.

The absence of observed HBV reactivation in our cohort is reassuring but should be interpreted cautiously, given that HBV reactivation has been reported during immune checkpoint inhibitor therapy and the overall risk depends on baseline viral status, viral load, and the use of antiviral prophylaxis and monitoring (14, 44). Accordingly, HBV management should be considered an integral co-intervention in HBV-positive patients receiving ICIs and/or TKIs, in line with oncology and hepatology guidance recommending systematic screening, antiviral prophylaxis, and longitudinal HBV DNA/alanine aminotransferase surveillance during cancer therapy (14, 44–46). In addition, our multivariable analysis identified baseline tumor diameter (≥ 7 cm) as an independent prognostic factor for both OS and PFS after matching, underscoring that tumor burden remains a central driver of outcomes even under intensified systemic and locoregional combination therapy (47, 48). This discovery strengthens the necessity of early identification and timely reassessment of resectability during treatment, as well as antiviral therapy and dynamic monitoring of viral load during treatment (10, 46, 48).

Several limitations warrant emphasis. The retrospective, single-center design introduces unavoidable selection bias and residual confounding, and the modest post-PSM sample size may have limited the statistical power of subgroup analyses and the stability of effect estimates. In addition, treatment heterogeneity existed because different PD-1 inhibitors were used; although this reflects real-world practice, it complicates attribution of differential effects. Another important limitation is that efficacy was assessed mainly using conventional radiologic criteria, without incorporation of tumor-immune biomarkers or advanced imaging-based approaches. Specifically, we did not profile biomarkers such as PD-L1 expression, immune cell infiltration, or angiogenic signatures, nor did we include imaging biomarkers or dynamic image-based modeling. Recent studies suggest that radiomics, longitudinal body composition analysis, and AI-assisted imaging evaluation may provide complementary noninvasive information on tumor heterogeneity, host condition, and treatment response in HCC treated with combination therapy, with potential value for earlier efficacy assessment, more refined patient stratification, and optimization of the timing of conversion surgery (49, 50). However, these emerging approaches still require prospective validation and methodological standardization before routine clinical implementation. Future multicenter prospective studies should therefore pre-specify HBV status as a stratification variable, incorporate standardized antiviral management, and integrate multimodal biomarker panels spanning virology, tumor genomics, immune-vascular profiling, and advanced imaging-based analyses. Such designs may better clarify whether HBV status independently modifies treatment effect or instead serves as a correlate of other determinants of response and conversion.

## 5 Conclusion

In this real-world cohort of patients with unresectable HCC undergoing triple conversion therapy, HBV-positive status was associated with higher surgical conversion rates and more favorable survival outcomes than HBV-negative status. This suggests that HBV status may serve as a potential stratification factor for treatment, although further prospective studies are still needed for validation.

## Data Availability

The original contributions presented in the study are included in the article/**Supplementary Material**, further inquiries can be directed to the corresponding author/s.
