# Supplementary material for: Hepatitis B virus infection and outcomes of TACE plus lenvatinib and PD-1 inhibitor therapy in unresectable hepatocellular carcinoma: a real-world propensity score-matched study
Source: Front Immunol. 2026 Apr 23;17:1808956. doi: 10.3389/fimmu.2026.1808956 (PMC13149396; doi:10.3389/fimmu.2026.1808956)
Supplement: Supplementary file 1 [file Supplementaryfile1.docx]

**Supplementary Table S1. Proposed reproducible criteria for successful conversion to resectability after triple conversion therapy for initially unresectable hepatocellular carcinoma**

| **Domain** | **Criterion** | **Recommended reproducible standard for study protocols** |
| --- | --- | --- |
| **Multidisciplinary decision** | MDT confirmation | **Required**. Resection should be approved by a multidisciplinary team including hepatobiliary surgeons, interventional radiologists, medical oncologists, and hepatologists. |
| **Surgical radicality** | Feasibility of curative resection | **Required**. Curative-intent **R0 resection** should be considered achievable. |
| **Future liver remnant (FLR)** | Adequate residual liver volume | **Required**. Suggested protocol threshold: **≥35%** in non-cirrhotic liver and **≥45%** in cirrhotic liver. |
| **Hepatic functional reserve** | Liver function suitable for hepatectomy | **Required**. Preferably **Child–Pugh A**; carefully selected **Child–Pugh B** may be considered in consensus-based practice, but most conversion studies use **Child–Pugh A only**. |
| **Performance status** | General condition | **Required**. **ECOG PS 0–1**. No major anesthesia or surgical contraindication. |
| **Tumor response: intrahepatic disease** | On-treatment disease control adequate for surgery | **Required**. Recommended unified definition: **complete response **(CR)** and **partial response (PR)**, or **stable disea**se (SD) maintained for at least 2 months**, provided all other resectability criteria are met. |
| **Tumor biology observation window** | Stability before surgery | **Recommended**. For technically resectable but biologically aggressive disease, consider requiring **tumor stability for 3–4 months** before resection. |
| **Vascular invasion** | PVTT/HVTT | **Required**. Tumor thrombus must become **completely resectable/removable** with preserved or reconstructable inflow/outflow. |
| **Extrahepatic disease** | Metastatic lesions | **Required**. No **unresectable active** extrahepatic disease at the time of surgery. |
| **Anatomical feasibility** | Vascular inflow/outflow and biliary drainage | **Required**. The remnant liver must have **preserved or reconstructable inflow, outflow, and biliary drainage**. |
| **Treatment safety** | Toxicity profile | **Required**. No **severe persistent treatment-related adverse events** or unresolved complications that would preclude hepatectomy. |
| **Perioperative timing** | Reassessment interval | **Recommended**. Reassess resectability every **6–8 weeks** or after every **2 treatment cycles**, depending on the protocol and TACE interval. |
| **Definition of successful conversion** | Endpoint for protocol use | **Proposed unified definition**: successful conversion is declared only when **all mandatory criteria above are fulfilled simultaneously** and MDT confirms that curative-intent hepatectomy can be performed safely. |

Abbreviations: MDT, multi-disciplinary team; ECOG, Eastern Cooperative Oncology Group; PVTT, portal vein tumor thrombus; HVTT, Hepatic vein tumor thrombus; TACE, Transcatheter arterial chemoembolization.
